# Proactive personality and career adaptability as predictors of clinical practice behavior among Chinese practical nursing students: the mediating role of career adaptability

**DOI:** 10.3389/fpubh.2026.1935627

**Published:** 2026-08-26

**Authors:** Qihui Han, Zhen Chen

**Affiliations:** 1 School of Nursing, Anhui Institute of Medicine, Hefei, Anhui, China; 2 School of Arts and Education, Chizhou University, Chizhou, Anhui, China

**Keywords:** career adaptability, career success, clinical practice behavior, mediation effect, nursing students, proactive personality, social cognitive career theory

## Abstract

**Background:**

This study aimed to examine the relationships among proactive personality, career adaptability, and clinical practice behavior among practical nursing students.

**Methods:**

A descriptive-correlational, cross-sectional design was employed with 394 practical nursing students. Data were collected using the Proactive Personality Scale, the Career Adapt-Abilities Scale—China Form, and the Clinical Evaluation Tool. Pearson correlation, hierarchical regression, and bootstrapping mediation analysis were conducted.

**Results:**

Proactive personality (*M* = 5.26, *SD* = 0.819), career adaptability (*M* = 3.74, *SD* = 0.535), and clinical practice behavior (*M* = 3.97, *SD* = 0.576) were all at moderately high levels. Significant positive correlations were found among all three variables. Career adaptability significantly mediated the association between proactive personality and clinical practice behavior, with an indirect effect of 0.5430, 95% CI [0.3978, 0.7229], accounting for 60.7% of the total effect.

**Conclusion:**

Career adaptability partially mediates the relationship between proactive personality and clinical practice behavior. Nursing education should focus on cultivating proactive personality and strengthening career adaptability to improve clinical practice behavior. However, longitudinal and multi-source studies are needed to validate the proposed pathway.

## 1 Introduction

According to the “State of Global Nursing 2020 Report” released by the World Health Organization (1), there were currently nearly 28 million nurses worldwide; however, a shortfall of 5.9 million nursing staff remains, with most gaps in Africa, Southeast Asia, the Eastern Mediterranean, and parts of Latin America. The Americas had the highest density of nurses at 83.4 per 10,000 people, followed by Europe with 79.3 per 10,000. In contrast, Africa had only 8.7 nurses per 10,000 people, the Eastern Mediterranean region had 15.6, Southeast Asia had 16.5, and the Western Pacific had 36 (1).

According to the “Statistical Bulletin on the Development of Chinese Health Undertakings in 2022” issued by the National Health Commission (2), by the end of 2022, there were 5.224 million registered nurses in China, with 37.1 registered nurses per 10,000 people, higher than in 2021. According to the “14th Five-Year Plan” Health Talent Development Plan, by 2025, the total number of registered nurses per 10,000 people would reach 38 (2). However, this has not reached the nurse population density of developed countries in Europe and the United States.

In addition, the turnover intention and turnover rate of nurses was high, especially among new nurses. The turnover intention of new nurses in the first year of employment in Asian countries was about 60% (3), in Europe about 33% (4), and in Turkey about 42.5% (5). A longitudinal study in South Korea showed that the turnover rate of new nurses in the first year was 7.3% (6). About 72% of new nurses in China planned to leave within the first year of employment, and the turnover rate of new nurses within the first 2 years was 4.45% (7). Increasing turnover of new nurses not only exacerbated the shortage of nursing staff but also hindered the sustainable development of nursing (8).

According to surveys, about 46% of new nurses chose to leave because of adjustment difficulties at the beginning of their careers (9). The early stage of a career is an important stage for new nurses to adapt, adjust, and transform their roles. At this stage, new nurses face difficulties and challenges from various aspects, resulting in poor adaptation, transition shock, work adaptation barriers, and subsequently having the idea of resignation or directly choosing to quit (3, 5, 9, 10).

Studies have shown that career adaptability can promote the transition and adaptation of new nurses from nursing students to nurses and reduce turnover intention (7, 11). Career adaptability referred to an individual’s readiness and coping ability when facing changes in the career process. Previous studies have shown that career adaptability can not only promote the career role transformation of nursing students but also effectively predict the level of clinical practice behavior of nursing students (12, 13). Clinical practice behaviors of nursing students refer to a series of behaviors in the clinical practice period, composed of knowledge, skills, emotion, motivation, and self-perception, and are the external manifestations of their clinical practice ability. Nursing students’ clinical practice behavior directly affects nursing quality and patient safety, as well as their future career development, and is a key indicator of the quality of higher nursing education and clinical teaching evaluation (14). Therefore, as a nursing educator, the researcher would like to pay attention to the development of students’ career adaptability and clinical practice behavior to help nursing students complete their career adaptation after graduation and promote their career success.

Clinical practice was the necessary learning stage for nursing students to transform from students to clinical nurses, the first step for nursing students to enter clinical nursing work before graduation, and the key link for nursing students to change from the role of students to the role of nurses, and an important transition period for their career running-in (15). However, due to the high intensity of clinical work, unfamiliarity with the clinical learning environment, obstacles in the transformation of theoretical knowledge into clinical practice, and role orientation, nursing students are prone to continuous pressure (16), and failure to adapt to this situation will not only affect the physical and mental health of a nursing student but even affect their intention to continue to engage in a nursing career (17).

In recent years, with the rapid development of positive psychology, researchers have paid more attention to proactive personality. A proactive personality is a kind of psychological tendency in which people take the initiative to seek solutions when facing difficulties. Studies have shown that a proactive personality has an important impact on individual adaptability (18). Other studies have shown that proactive personality plays an important role in establishing good interpersonal relationships, reducing nurse–patient conflicts, and improving nursing service and performance (19). The research of Chen et al. indicated that we should pay more attention to cultivating nurses’ proactive personalities and improving nurses’ innovative levels (20).

The present study was theoretically informed by Social Cognitive Career Theory (SCCT) and Career Construction Theory. In SCCT, career-related behaviors are shaped by the interaction among person inputs, learning experiences, self-regulatory resources, goals, and contextual factors. Proactive personality can be conceptualized as a relatively stable person input or dispositional resource that encourages individuals to identify opportunities, take initiative, and actively shape their learning and work environments. Career adaptability, which originates from Career Construction Theory, represents a set of psychosocial resources that enable individuals to cope with current and anticipated career tasks, transitions, and challenges. Clinical practice behavior can be regarded as a behavioral performance outcome in the clinical learning context. Therefore, proactive personality may be positively associated with clinical practice behavior both directly and indirectly through the development of career adaptability. However, given the cross-sectional design of this study, the proposed pathway should be interpreted as a conceptual and heuristic model rather than as evidence of causal ordering.

This study intended to investigate the status quo of proactive personality, career adaptability, and clinical practice behavior of nursing students, and the relationship among the three variables in order to provide a basis for career planning and humanistic values education among nursing students.

## 2 Materials and methods

### 2.1 Research design

This study adopted a descriptive-correlational, cross-sectional design. This design was appropriate because the study aimed to describe the current levels of proactive personality, career adaptability, and clinical practice behavior and to examine the statistical associations among these variables. Mediation analysis was used to test whether career adaptability statistically accounted for part of the association between proactive personality and clinical practice behavior. Because all data were collected at one time point, the mediation model was interpreted as an associational model rather than as evidence of causal mediation.

### 2.2 Research locale

The study was conducted in a medical college located in Hefei City, Anhui Province, China. It is a public general college directly under the Anhui Provincial Health Commission. It consists of the Basic Medicine Institution, Public Basic Science Institution, Marxism Institution, Nursing Institution, Stomatology Institution, Clinical Medicine Institution, Medical Technology Institution, Public Health and Health Management Institution, Pharmacy Institution, International Education Institution, and Continuing Education Institution, with 18 majors, with a total of more than 12,000 students, including more than 2,200 students in the nursing institution, and more than 700 students participated in clinical practice after finishing theoretical study in February 2024. The cooperative practice hospitals of the Nursing Institution are distributed in various provinces and cities across the country, with a total of 55, all of which are first-class (Grade-III) general hospitals.

### 2.3 Participants

The population for this study consisted of practical nursing students enrolled in a medical college in China. Systematic sampling was employed to select participants. The procedure was as follows: (1) a list of all practical nursing students enrolled in the medical college was compiled; (2) the total population size was determined; (3) a sampling interval (k) was calculated by dividing the total population size by the desired sample size; (4) a random start was chosen between 1 and k; (5) subsequently, every kth participant was selected until the desired sample size was reached.

The required sample size was estimated using G*Power for multiple linear regression. With two predictors, a medium effect size of *f^2^* = 0.15, *α* = 0.05, and statistical power = 0.95, the minimum required sample size was approximately 107. Considering the need for stable parameter estimation in mediation analysis and potential invalid responses, a larger sample was targeted. A total of 394 practical nursing students were recruited, which exceeded the minimum requirement and provided adequate statistical power for correlation, regression, and mediation analyses.

Inclusion criteria: (a) practical nursing students enrolled in the medical college in China; (b) students who have not yet finished clinical practice; (c) students without any cognitive disorders; (d) students who provide informed consent to participate in the study.

Exclusion criteria: (a) students with cognitive disorders.

Withdrawal criteria: participants can withdraw at any time.

### 2.4 Research instruments

Four instruments were used in this study.

The first instrument is the demographic profile, consisting of age, gender, and sib ling rank.

The second instrument is the Proactive Personality Scale (PPS), developed by Bateman and Crant (21). In this study, the researcher used the PPS revised by Shang and Gan (22), which is suitable for Chinese populations. The scale consisted of 11 items with a single-dimensional structure, and a seven-point Likert scale was adopted, with 1–7 indicating “strongly disagree” to “strongly agree.” The higher the score, the greater the corresponding proactive personality tendency. The internal consistency reliability of the scale is 0.90, and the split-half reliability is 0.85. The Cronbach’s alpha coefficient of the scale as a whole is 0.779.

The third instrument was the Career Adapt-Abilities Scale (23)—China Form (CAAS-China Form), based on the Career Adapt-Abilities Scale developed by Savickas and Porfeli and translated and validated in Chinese by Hou et al. (24). The scale contains 24 items across four dimensions: career concern (items 1–6), career control (items 7–12), career curiosity (items 13–18), and career confidence (items 19–24), with six items in each dimension. A 5-point Likert scoring method is adopted. Higher scores indicate stronger career adaptability. The Cronbach’s *α* coefficient of the overall scale was 0.899, and the Cronbach’s α coefficients of the four subscales were 0.746, 0.755, 0.767, and 0.872, respectively. The 24-item CAAS-China Form was used rather than the 12-item short form. This clarification is important because long and short forms of career adaptability measures may differ in their criterion-related validity and in the magnitude of their associations with other psychological and behavioral constructs (25).

The fourth instrument is the Clinical Evaluation Tool (CET) developed by Deng (26), including 3 first-level indicators in cognitive, emotional, and operational skills, 7 s-level indicators, and 28 third-level indicators. The cognitive field included the application of theoretical knowledge (items 1–3) and the application of nursing procedures (items 23–28). The emotional field included professional roles and role development (items 4–8), self-directed learning behaviors (items 9–12), and clarifying patients’ rights and obligations (items 13–15). The operational skills field included clinical skills (items 16–18) and communication skills (items 19–22). Using a 5-level scoring system, “5″ means always, “4″ means often, “3″ means sometimes, “2″ means rarely, and “1″ means never. The total score ranges from 28 to 140 points; the higher the score, the better the clinical practical behavior. The item consistency, content validity, Cronbach’s *α* coefficient, and split-half reliability of the scale were 0.89, 0.88, 0.93, and 0.81, respectively.

### 2.5 Pilot test

The researcher used convenience sampling to select 50 participants who met the inclusion criteria and exclusion criterion (no cognitive disorders) from Anhui Medical College for a pilot test. The Cronbach’s alpha coefficient of PPS as a whole was 0.745. The Cronbach’s *α* coefficient of the CAAS-China Form as a whole was 0.792. The Cronbach’s α coefficient of CET as a whole was 0.813. All three scales used in this study had good reliability and validity.

### 2.6 Ethical considerations

This study underwent approval from the Ethics Review Committee of St. Paul University. The researcher followed ethical principles of beneficence, respect for human dignity, and justice. Informed consent was secured from all participants. Participants had the right to withdraw at any time. Information was kept anonymous and confidential.

### 2.7 Data analysis procedure

Descriptive statistics were used to summarize demographic characteristics and the levels of proactive personality, career adaptability, and clinical practice behavior. For Tables 1–3, item-level composite means were reported to facilitate interpretation of participants’ average responses on each scale. For correlation and regression analyses, total scores were used unless otherwise specified.

**Table 1:** Perception of practical nursing students in terms of proactive personality (*N* = 394).

Item	*Mean*	*SD*	Interpretation
(1) If I see someone in trouble, I will try my best to help.	5.70	1.202	Good
(2) I’m good at turning problems into opportunities.	4.86	1.210	Moderately Good
(3) I’m always looking for a better way to do things.	5.63	1.105	Good
(4) When I have a problem, I face it.	5.29	1.191	Moderately Good
(5) I like to challenge the status quo.	4.66	1.288	Moderately Good
(6) What if I believe in an idea and there are no obstacles that can stop me from realizing it?	4.82	1.275	Moderately Good
(7) If I believe in something, I’ll do it regardless of the odds.	5.23	1.200	Moderately Good
(8) There’s nothing more exciting than seeing my ideas come to life.	5.76	1.177	Good
(9) I’m always looking for new ways to make my life better.	5.74	0.998	Good
(10) I enjoy facing and overcoming obstacles to my ideas.	5.08	1.288	Moderately Good
(11) I always wanted to be special in my community (and maybe in the world).	5.06	1.284	Moderately Good
Composite Mean	5.26	0.819	Moderately Good

1.00–1.85 = Very Poor; 1.86–2.71 = Poor; 2.72–3.57 = Moderately Poor; 3.58–4.43 = Neutral; 4.44–5.29 = Moderately Good; 5.30–6.15 = Good; 6.16–7.00 = Very Good.

**Table 2:** Perception of practical nursing students in terms of career adaptability (*N* = 394).

Item	*Mean*	*SD*	Interpretation
(1) Think about what my future will be like.	3.57	0.813	Moderately Strong
(2) Know the choices I make now will shape my future.	3.70	0.850	Moderately Strong
(3) Prepare for the future.	3.64	0.789	Moderately Strong
(4) Know the educational and career choices I have to make.	3.69	0.835	Moderately Strong
(5) Plan to achieve my goals.	3.55	0.802	Moderately Strong
(6) Pay attention to career.	3.70	0.871	Moderately Strong
(7) Keep an optimistic attitude.	3.82	0.886	Moderately Strong
(8) Make my own decisions.	3.74	0.814	Moderately Strong
(9) Take responsibility for my actions.	4.01	0.716	Moderately Strong
(10) Stick to your beliefs.	3.74	0.784	Moderately Strong
(11) Rely on myself.	3.81	0.776	Moderately Strong
(12) Do what’s right for myself.	3.95	0.747	Moderately Strong
(13) Explore my surroundings.	3.72	0.795	Moderately Strong
(14) Look for opportunities to grow.	3.77	0.807	Moderately Strong
(15) Consider all possible options before making a decision.	3.90	0.823	Moderately Strong
(16) Observe different ways of doing things.	3.75	0.846	Moderately Strong
(17) Explore deeply into the issues I care about.	3.82	0.762	Moderately Strong
(18) Be curious about new opportunities.	3.83	0.769	Moderately Strong
(19) Perform tasks efficiently.	3.51	0.882	Moderately Strong
(20) Take things seriously.	3.87	0.767	Moderately Strong
(21) Learn new skills.	3.72	0.769	Moderately Strong
(22) Gradually develop my ability.	3.79	0.773	Moderately Strong
(23) Overcome obstacles.	3.59	0.812	Moderately Strong
(24) Solve the problem.	3.63	0.834	Moderately Strong
Composite Mean	3.74	0.535	Moderately Strong

*N* = 394. 1.00–1.80 = Weak; 1.81–2.60 = Moderately Weak; 2.61–3.40 = Middle; 3.41–4.20 = Moderately Strong; 4.21–5.00 = Strong.

**Table 3:** Perception of practical nursing students in terms of clinical practical behavior (*N* = 394).

Item	*Mean*	*SD*	Interpretation
(1) Use nursing theory to guide clinical practice. (e.g., hierarchy of needs, self-protection theory).	3.66	0.825	Good
(2) Apply the theoretical knowledge of related disciplines to guide clinical practice.	3.70	0.799	Good
(3) Collect information on discipline development and apply it to clinical practice.	3.48	0.910	Good
(4) Establish and implement personal professional development goals.	3.58	0.909	Good
(5) Conduct yourself in accordance with the requirements of the nurse role.	3.82	0.884	Good
(6) Treat patients equally.	4.29	0.728	Good
(7) Providing sound advice to patients and their families (lead role).	3.93	0.881	Good
(8) Coordinate the relationship between relevant personnel to solve the problems of patients (inquire about the cost for patients, inquire about the treatment plan, etc.)	3.94	0.863	Good
(9) Learn actively based on your own or others’ evaluation.	3.82	0.860	Good
(10) Participate in academic activities organized by the hospital or department.	3.62	0.962	Good
(11) Explore and solve problems in nursing work.	3.78	0.866	Good
(12) Reflect on your work during the internship.	3.96	0.819	Good
(13) Respect the patient’s choice of treatment and nursing (e.g., the patient refuses to give an infusion to the student nurse).	3.93	0.918	Good
(14) Explain and explain to the patient before performing nursing procedures. (Clarify the patient’s right to know)	4.21	0.790	Very Good
(15) Protect patient privacy.	4.51	0.699	Very Good
(16) Practice to implement basic nursing operations.	4.16	0.730	Good
(17) Skilled implementation of specialized nursing operations.	4.14	0.795	Good
(18) Skilled use of commonly used medical instruments and equipment.	4.01	0.808	Good
(19) Evaluate who you are communicating with and use targeted communication methods.	4.08	0.781	Good
(20) Communicate information clearly to the person you are communicating with. (Spoken language)	4.20	0.740	Good
(21) Adjust the style and content of the communication according to the response of the audience.	4.13	0.749	Good
(22) Records are concise, accurate, neatly written, and medical terminology is used.	4.11	0.816	Good
(23) Collect information about the patient’s health.	4.10	0.826	Good
(24) Identify existing and potential health problems.	4.04	0.802	Good
(25) Develop individualized care goals.	3.93	0.868	Good
(26) Implement nursing measures in line with nursing behavior norms.	4.10	0.781	Good
(27) Evaluation of nursing effect.	4.05	0.820	Good
(28) Revised care plan.	3.94	0.851	Good
Composite Mean	3.97	0.576	Good

*N* = 394. 1.00–1.80 = Very Poor; 1.81–2.60 = Poor; 2.61–3.40 = Neutral; 3.41–4.20 = Good; 4.21–5.00 = Very Good.

Before testing the structural associations, the measurement properties of the instruments were examined. Internal consistency was assessed using Cronbach’s alpha and composite reliability. Confirmatory factor analysis was conducted to evaluate construct validity. Convergent validity was assessed using standardized factor loadings and average variance extracted. Discriminant validity was examined using the Fornell–Larcker criterion and, where applicable, the heterotrait–monotrait ratio.

Common method bias was assessed because all variables were collected from the same respondents at the same time using self-report questionnaires. Harman’s single-factor test was conducted, and the percentage of variance explained by the first unrotated factor was examined. In addition, full collinearity variance inflation factors were calculated as a supplementary diagnostic.

Pearson’s correlation coefficients were used to examine bivariate associations among proactive personality, career adaptability, and clinical practice behavior. Hierarchical regression analysis was conducted to examine the mediating role of career adaptability in the association between proactive personality and clinical practice behavior. Both unstandardized coefficients and standardized coefficients were reported. Bootstrapping analysis with 5,000 resamples was used to test the indirect effect. Mediation was considered significant when the 95% confidence interval of the indirect effect did not include zero. Effect sizes, including *R^2^*, adjusted *R^2^*, *ΔR^2^*, and *f^2^*, were also reported.

## 3 Results

### 3.1 Demographic profile of respondents

Table 4 presented the demographic profile of the respondents. In terms of age, the majority were 20 years old (40.10%), followed by 19 years old (35.53%) and 21 years old (24.37%). Regarding gender, the majority were female (88.07%) compared to male respondents (11.93%). In sibling rank, the data showed that a significant number of respondents were second-born (53.30%), followed by third-born (23.10%), only children (13.45%), and fourth-born onwards (10.15%).

**Table 4:** Demographic profile of the respondents (*N* = 394).

Profile of Students	Frequency	Percentage
	*n*	%
Age		
19 yrs. old	140	35.53%
20 yrs. old	158	40.10%
21 yrs. old	96	24.37%
Gender		
Male	47	11.93%
Female	347	88.07%
Sibling Rank		
Only Child	53	13.45%
Second	210	53.30%
Third	91	23.10%
Fourth Onwards	40	10.15%
Total	394	100.0%

### 3.2 Perception of respondents on proactive personality, career adaptability, and clinical practice behavior

Table 1 showed the mean and standard deviation distribution of respondents for proactive personality traits. The composite mean score was 5.26 (*SD* = 0.819), interpreted as “Moderately Good.” Item 8 (“There’s nothing more exciting than seeing my ideas come to life”) generated the highest mean score (*M* = 5.76, *SD* = 1.177), while item 5 (“I like to challenge the status quo”) produced the lowest mean (*M* = 4.66, *SD* = 1.288).

Table 2 depicted the results for career adaptability. The composite mean score was 3.74 (*SD* = 0.535), interpreted as “Moderately Strong.” Item 9 (“Take responsibility for my actions”) obtained the highest mean score (*M* = 4.01, *SD* = 0.716), and item 19 (“Perform tasks efficiently”) garnered the lowest mean score (*M* = 3.51, *SD* = 0.882).

Table 3 illustrates the practical nursing students’ perceptions regarding their clinical behavior. The overall mean was 3.97 (*SD* = 0.576), interpreted as “Good.” Item 15 (“Protect patient privacy”) generated the highest mean score (*M* = 4.51, *SD* = 0.699), while item 3 (“Collect information on discipline development and apply it to clinical practice”) produced the lowest mean score (*M* = 3.48, *SD* = 0.910).

### 3.3 Correlation analysis

Table 5 shows the correlation matrix among proactive personality, career adaptability, and clinical practice behavior. Significant positive correlations were observed among all variables. Proactive personality and clinical practice behavior were significantly positively correlated (*r* = 0.500, *p* < 0.001). Proactive personality and career adaptability were significantly positively correlated (*r* = 0.623, *p* < 0.001). Career adaptability and clinical practice behavior were significantly positively correlated (*r* = 0.610, *p* < 0.001).

**Table 5:** Correlation analysis of clinical practical behavior, career adaptability, and proactive personality (*N* = 394).

Variable	*M*	*SD*	1	2	3
Proactive Personality	57.83	9.026	–		
Career Adaptability	89.82	12.861	0.623***	–	
Clinical Practical Behavior	111.21	16.144	0.500***	0.610***	–

Means and standard deviations in this table are based on total scores, whereas Tables 1–3 report item-level composite means for ease of interpretation. PP = proactive personality; CA = career adaptability; CPB = clinical practice behavior. ***p < 0.001.

### 3.4 Mediation analysis

Table 6 presents the hierarchical regression results. The coefficients previously reported as *β* were rechecked and identified as unstandardized regression coefficients. Therefore, both unstandardized coefficients and standardized coefficients are reported in the revised table. In Model 1, proactive personality was positively associated with career adaptability, *B* = 0.876, standardized *β* = 0.615, *p* < 0.001. In Model 2, proactive personality was positively associated with clinical practice behavior, *B* = 0.885, standardized β = 0.495, *p* < 0.001. In Model 3, career adaptability was positively associated with clinical practice behavior, *B* = 0.762, standardized β = 0.607, *p* < 0.001. In Model 4, when proactive personality and career adaptability were entered simultaneously, both remained significant correlates of clinical practice behavior. The standardized coefficients were β = 0.194 for proactive personality and β = 0.484 for career adaptability. The final model explained approximately 39.4% of the variance in clinical practice behavior, indicating a moderate-to-large effect.

**Table 6:** Hierarchical regression analysis results of variables (*N* = 394).

PV	Career adaptability		Clinical practical behavior					
DV								
	Model 1		Model 2		Model 3		Model 4	
	*B*	*β*	*B*	*β*	*B*	*β*	*B*	*β*
Constant	11.861		36.476		36.234		29.267	
Gender (Male = 2, Female = 1)	−1.106	−0.028	−1.621	−0.033	−1.082	−0.022	−0.949	−0.019
Sibling Rank	0.32	0.002	0.603	0.031	0.508	0.026	0.583	0.030
Age	1.433^*^	0.085	1.235	0.059	0.357	0.017	0.364	0.017
Proactive Personality	0.876^***^	0.615	0.885^***^	0.495			0.353^***^	0.194
Career adaptability					0.762^***^	0.607	0.608^***^	0.484
*R* ^2^	0.395		0.256		0.374		0.398	
*F*	63.602^***^		33.423^***^		58.027^***^		51.201^***^	
*△R* ^2^							0.142	
*△F*							91.285^***^	

^*^p < 0.05, ^***^p < 0.001. PV = predictor variable; DV = dependent variable.

**Figure 1:**
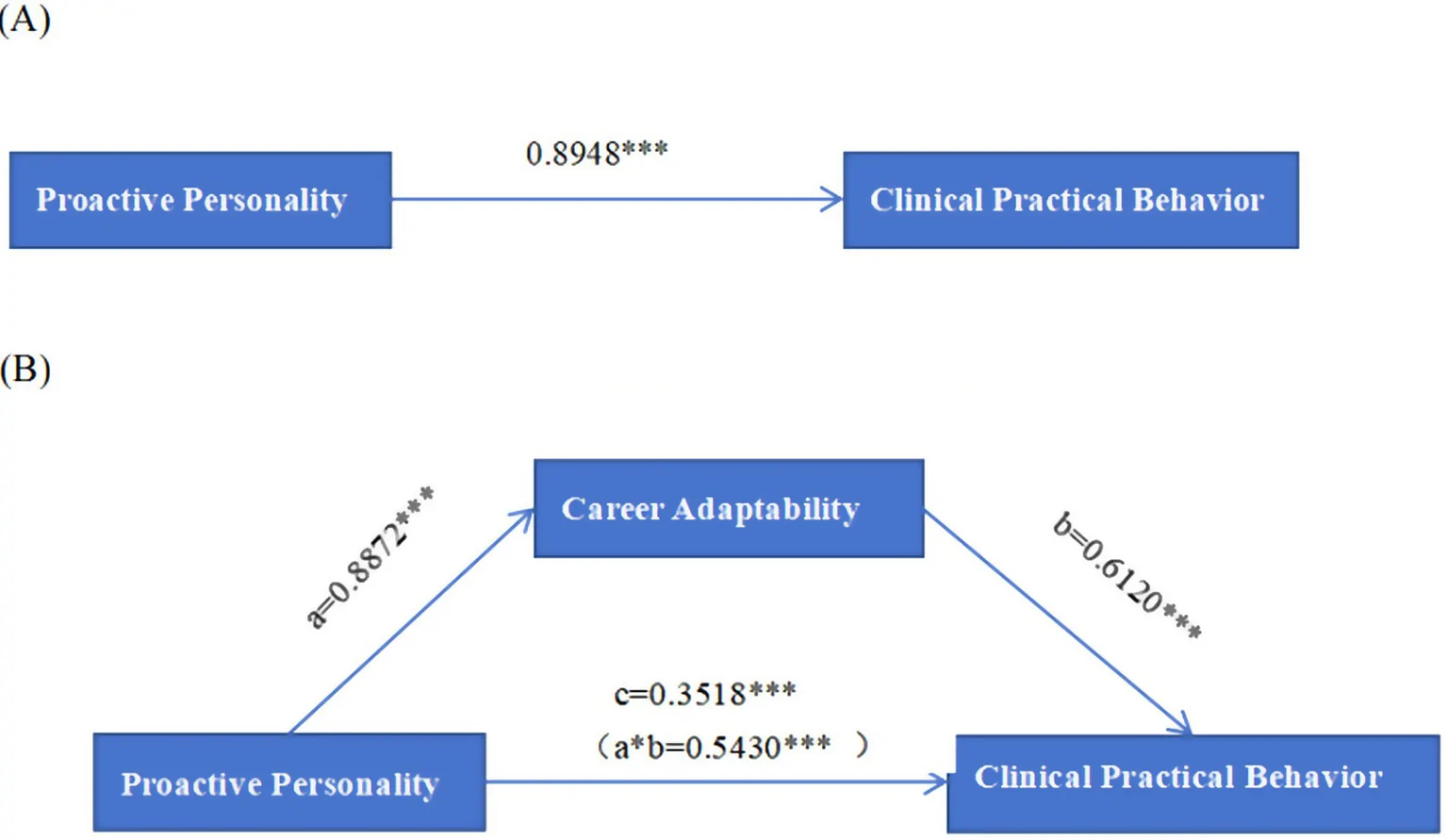
Mediation model results. **(A)** Total effect model. **(B)** Direct and indirect effect model. ***p < 0.001.

The bootstrapping analysis showed that the total effect of proactive personality on clinical practice behavior was significant, effect = 0.8948, *p* < 0.001, 95% *CI* [0.7410, 1.0486]. The direct effect remained significant, effect = 0.3518, *p* < 0.001, 95% *CI* [0.1752, 0.5285]. The indirect effect through career adaptability was also significant, effect = 0.5430, 95% *CI* [0.3978, 0.7229], accounting for 60.7% of the total effect. These findings suggest that career adaptability partially mediated the association between proactive personality and clinical practice behavior. Because the data were cross-sectional, this mediation should be interpreted statistically rather than causally. These results are visually presented in Figure 1.

## 4 Discussion

This study investigated the relationship between proactive personality, career adaptability, and clinical practice behavior among practical nursing students in China. The findings revealed that all three variables were at moderately high levels, with significant positive correlations among them. Most importantly, career adaptability was found to play a significant mediating role between proactive personality and clinical practice behavior.

The findings can be interpreted through the combined lens of SCCT and Career Construction Theory. Within SCCT, proactive personality may function as a person input that encourages students to actively seek learning opportunities, engage with clinical challenges, and regulate their own professional development. Career adaptability, derived from Career Construction Theory, represents adaptive resources that help students prepare for career tasks, make career-related decisions, explore possible professional roles, and maintain confidence in the face of clinical challenges. Clinical practice behavior reflects students’ behavioral engagement and performance in the clinical learning environment. Therefore, the positive association between proactive personality and clinical practice behavior may be partly explained by the role of career adaptability as an adaptive resource. Students with stronger proactive personality may be more likely to develop concern, control, curiosity, and confidence regarding their future nursing careers, which in turn may be associated with better clinical practice behavior.

In addition, this study used the 24-item CAAS-China Form rather than the short form of the Career Adapt-Abilities Scale. The use of the full version allowed a more comprehensive assessment of the four dimensions of career adaptability. Recent evidence suggests that long and short measures of career adaptability may differ in their criterion-related validity and may yield different magnitudes of association with career-related outcomes. Therefore, future studies may compare the full and short forms among nursing students to determine which version is more suitable for nursing education research and practice (25).

The composite mean score of proactive personality (*M* = 5.26) indicated a “Moderately Good” level among practical nursing students. This was consistent with previous studies in China (27, 28). The results suggest that practical nursing students generally possess positive and proactive orientations, which are crucial for their adaptation to the demanding clinical environment.

The composite mean score of career adaptability (*M* = 3.74) was “Moderately Strong,” similar to findings by Xu et al. (29) and Peng and Wang (30). This indicated that career development and planning education in Anhui medical colleges has achieved initial results. Under the current situation of increasingly serious employment pressure, most college practical nursing students who are before graduation have begun to pay attention to their own career development and can deal with their own career development problems independently.

The composite mean score of clinical practice behavior (*M* = 3.97) was “Good,” consistent with studies by Li et al. (31) and Zhou et al. (32). The practical nursing students have systematically learned the basic theory, basic knowledge, and basic skills of nursing. However, the ability to collect information on discipline development and apply it to clinical practice (item 3, *M* = 3.48) needs improvement, possibly due to the gap between theoretical teaching and clinical practice in college.

The significant positive correlation between proactive personality and clinical practice behavior (*r* = 0.500, *p* < 0.001) supports previous research by Wei and Yuan (27), who found that higher levels of proactive personality are associated with better self-regulation in clinical nursing practice. This suggests that nursing students with proactive personalities are more likely to engage in positive clinical behaviors.

The significant positive correlation between proactive personality and career adaptability (*r* = 0.623, *p* < 0.001) is consistent with multiple studies in China (27, 30, 33, 34). Individuals with a strong proactive personality are more inclined to proactively adapt to new environments, seize opportunities, and create conditions for career exploration and planning.

The significant positive correlation between career adaptability and clinical practice behavior (*r* = 0.610, *p* < 0.001) suggests that nursing students with higher career adaptability demonstrate better clinical practice behaviors. This may be because higher levels of career adaptability provide students with more career concern, career control, career curiosity, and career confidence, enabling them to adapt more easily to new clinical practice environments.

The mediation analysis revealed that career adaptability significantly mediated the relationship between proactive personality and clinical practice behavior, with an indirect effect ratio of 60.7%. This indicates that a substantial portion of the effect of proactive personality on clinical practice behavior operates through career adaptability. This finding is theoretically grounded in Social Cognitive Career Theory (SCCT) (35), which posits that individual background factors (such as proactive personality and career adaptability) interact with environmental factors to influence career-related behaviors.

**Figure 2:**
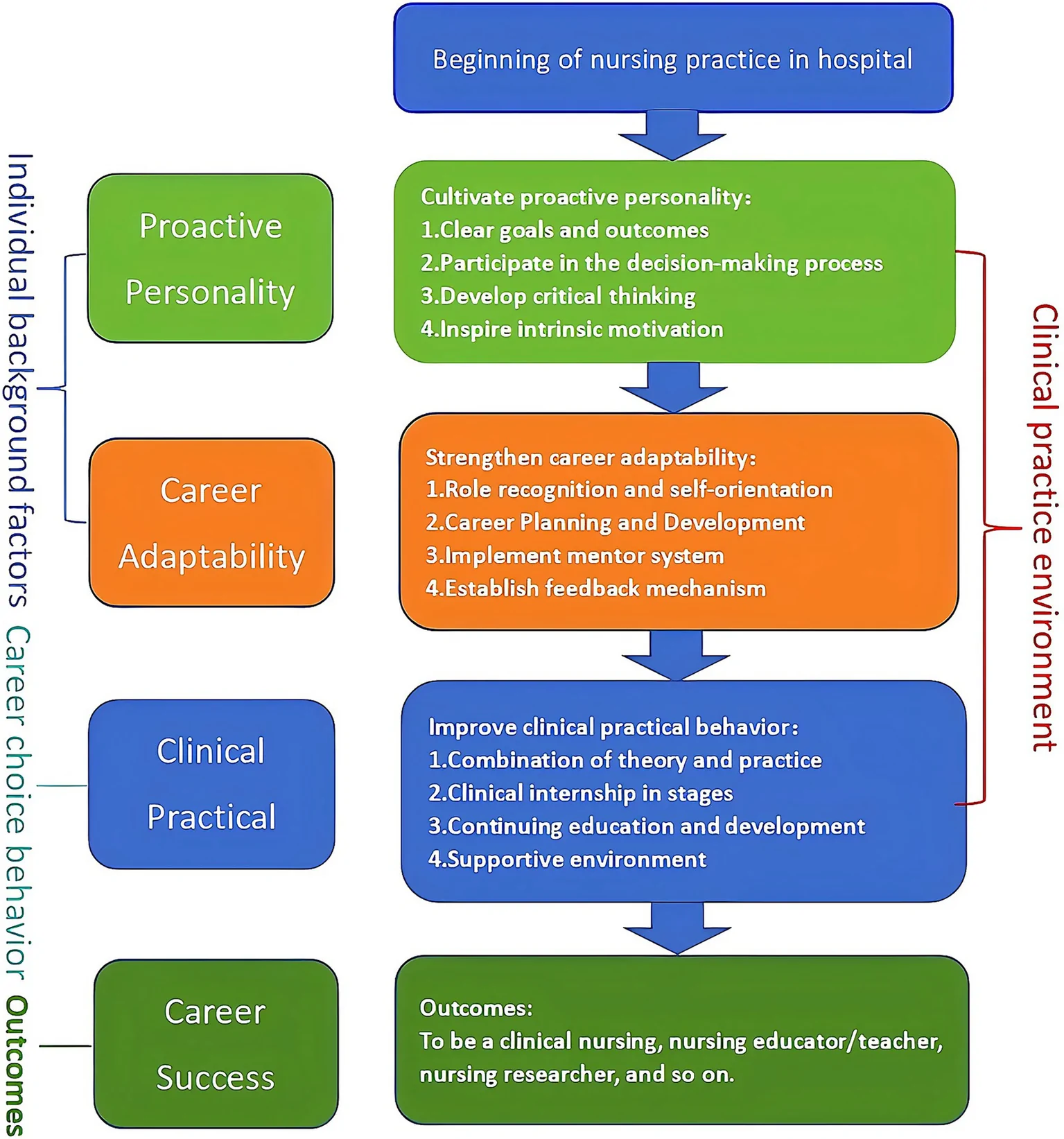
Career success path for practical nursing students.

Based on these findings, a career success path for practical nursing students is proposed (Figure 2). The roadmap encompasses three main components: (1) cultivating a proactive personality; (2) strengthening career adaptability; and (3) improving clinical practical behavior.

## 5 Limitations and future directions

The limitations were that the research only explored about the Proactive Personality, career adaptability, and Clinical Practical Behavior of Chinese practical nursing students and the relationship among the three variables, it might have factors that the researcher has not yet considered. And there was no perceptions of clinical instructors to the practical nursing students on the Proactive Personality, Career Adaptability, and Clinical Practical Behavior. And a small sample of practical nursing students included in this study, therefore may not represent all of the practical nursing students across China. In the future, the researcher could consider more factors and increase the sample, and verify that the intervention based the research is effective or not, to promote the career success of practical nursing students and solve the problem of shortfall nursing staff in China, even all over the word.

## 6 Conclusion

This study found that proactive personality, career adaptability, and clinical practice behavior were positively associated among Chinese practical nursing students. Career adaptability partially mediated the association between proactive personality and clinical practice behavior, suggesting that students with stronger proactive tendencies may report better clinical practice behavior partly through stronger career adaptability. The findings highlight the importance of cultivating proactive personality traits and strengthening career adaptability in nursing education. However, because this study was cross-sectional and based on self-report data from one college, the proposed career development pathway should be regarded as a conceptual model requiring further validation through longitudinal, multi-site, and multi-source research.

## Data Availability

The raw data supporting the conclusions of this article will be made available by the authors, without undue reservation.
